# From expert to novice and back: a qualitative study of interprofessional collaboration and the experiences of frontline healthcare professionals during the first wave of COVID-19

**DOI:** 10.1186/s12909-023-04262-9

**Published:** 2023-05-02

**Authors:** Malin Heiden, Camilla Bernild, Selina Kikkenborg Berg, Ilkay Dagyaran, Malene Missel, Signe Westh Christensen, Signe Stelling Risom, Ida Elisabeth Højskov

**Affiliations:** 1 Department of Infectious Diseases, The Heart Centre, Copenhagen University Hospital − Rigshospitalet, Ester Møllers Vej 6, Copenhagen, 2100 Denmark; 2 The Heart Centre, Copenhagen University Hospital − Rigshospitalet, Copenhagen, Denmark; 3 Department of Clinical Medicine, Faculty of Health and Medical Sciences, National Institute of Public Health, University of Southern Denmark, University of Copenhagen, Copenhagen, Denmark; 4 Department of Heart and Lung Surgery, The Heart Centre, Copenhagen University Hospital − Rigshospitalet, Copenhagen, Denmark; 5 The Heart Association, Copenhagen, Denmark; 6 Department of Clinical Medicine Faculty of Health and Medical Sciences, University of Copenhagen, Copenhagen, Denmark; 7 Institute of Nursing and Nutrition, University College Copenhagen, Copenhagen, Denmark; 8 Department of Cardiology, Herlev and Gentofte Hospital, Hellerup, Denmark

**Keywords:** COVID-19, Health Care professionals, Interprofessional relations, Learning transfer, Working environment, Qualitative research

## Abstract

**Background:**

The global coronavirus disease 2019 pandemic put extreme pressure on healthcare systems worldwide, forcing a heavy workload on healthcare professionals. Frontline treatment and care for patients with coronavirus disease 2019 compelled healthcare professionals to rapidly adapt to new working conditions. This study explores the experiences of frontline healthcare professionals to learn more about how frontline work affects their learning and skills development but also interprofessional collaboration during a pandemic.

**Methods:**

In-depth, one-to-one semi-structured interviews were conducted with 22 healthcare professionals. A broad interdisciplinary group, the participants were employed in public hospitals in four of Denmark’s five regions. Using a reflexive methodology for the data analysis allowed reflexive interpretation when interpreting subjects and interpreting the interpretation.

**Results:**

The study identified two empirical themes: into the unknown and in the same boat, which we critically interpreted using learning theory and theory on interprofessionalism. The study found that the healthcare professionals moved from being experts in their own fields to being novices in the frontline of the pandemic, and then back to being experts based on interprofessional collaboration that included shared reflection. Working in the frontline was imbued with a unique atmosphere in which workers were equals and functioned interdependently, the barriers normally obstructing interprofessional collaboration set aside to focus on combating the pandemic.

**Conclusions:**

This study reveals new insights regarding knowledge on frontline healthcare professionals in terms of learning and developing new skills, as well as the importance of interprofessional collaboration. The insights contributed to the understanding of the importance of shared reflection and how the development of expertise was a socially embedded process where discussions were possible without fear of being ridiculed and healthcare professionals were willing to share their knowledge.

## Background

The drastic changes that occurred in response to the coronavirus disease 2019 (COVID-19) pandemic have undoubtedly affected the nature of interprofessional interactions. Healthcare professionals (HCPs) worldwide continue to face a serious, unprecedented situation in which they are forced to make difficult decisions while under immense physical and psychological pressure. The uncertainty of the pandemic led to panic and confusion in the effort to fight the virus. As a result, there is an urgent need to explore the experiences and challenges HCPs in the frontline of the pandemic faced, but also to disseminate the lessons learned through first-hand experience.

First detected in China in December 2019, the coronavirus was declared a pandemic by the World Health Organization (WHO) on 11 March 2020 [1]. With national and international restrictions implemented to minimise the spread of the virus and to improve the treatment of patients who are infected, pandemics greatly impact people worldwide. Despite the measures taken the virus spread rapidly, resulting in such a high increase in patients admitted to the hospital that it threatened their capacity [1].

The first case of COVID-19 in Denmark was diagnosed in the end of February 2020 [2]. The health authorities aimed to contain the infection through strict isolation of cases and contact tracing. However the virus began to spread and the strategy changed from containment to mitigation [2, 3]. A temporary closedown of schools and childcare institutions was announced, and many employees were required to work from home. Hospitals prepared for an inflow of COVID-19 patients and outpatient visits and elective surgery were postponed. In the beginning of April, a very slow and gradual reopening was initiated [3, 4]. The Danish hospitals are run by five different regions. The Danish health care system free for everyone, independent of health insurance, and with a tradition of a large public sector of high-quality hospitals and clinics, with relatively few private clinics [5].

Disaster and war-torn areas, often the province of HCPs [6], are currently a common aspect of their work worldwide. With the seriousness of the COVID-19 situation quickly becoming apparent [1], its urgency called for simultaneously taking action and organising. Normally the healthcare system in Denmark is able to absorb a larger number of patients, but the seriousness of this previously unknown virus, combined with the influx of critically ill patients requiring isolation, put hospitals to the test in terms of the logistics involved to manage the pandemic [7, 8].

In addition it was necessary to organise and recruit HCPs for wards specially set up for treating COVID-19 [9]. Initially, HCPs with various specialties were needed to establish the new COVID-19 wards, forcing HCPs with different specialties to work together in an unfamiliar environment. The severity of the situation left little time to educate and train them, pushing them to familiarise themselves with new tasks and routines [10], some of which they were accustomed to from their daily clinical practice, while others were new and required the acquisition of new knowledge, skills, and abilities [11]. Thus, HCPs in the frontline of the pandemic worked in a highly complex and difficult situation, many of whom felt they were under enormous pressure, like soldiers at war. Changes in professional responsibilities, working hours, and shifts threatened their work-life balance [12]. Despite this highly difficult situation filled with uncertainty and unpredictability, the HCPs managed to provide treatment and care for patients with COVID-19. This study aims to learn more about how being in the frontline of COVID-19 affected learning and skills development among HCPs, but also their interprofessional collaboration.

## Methods

This study used a qualitative approach that included conducting in-depth, one-to-one semi-structured interviews with 22 HCPs who were frontline workers during the first wave of COVID-19 to gain insight into how being in the frontline affected their learning and skills development and interprofessional collaboration.

### Sampling and participants

To ensure a wide range of HCPs in terms of age, sex, profession, and degree of clinical experience, the participants were recruited using purposeful sampling and convenience sampling [13] among a population of HCPs who were on a COVID-19 ward. An administrative employee from the hospital who was not involved in the study approached the COVID-19 wards in the five different regions of Denmark to find eligible informants. We selected key informants with rich information on the research area based on individual judgment, the subject, and the following question: Who can provide sufficient, relevant, and deep information about the topic? The inclusion criteria were appropriate experience with caring for COVID patients, willingness to participate, and willingness to share experiences. The exclusion criteria were no experience in caring for and treating patients with COVID-19. Our aim was for the participants to represent a broad professional group across specialties and with various levels of experience. In total we reached out to 26 HCP but 4 of them could not participate in the study for unknown reasons. All participants worked at public hospitals in four of Denmark’s five regions and comprised a broad interdisciplinary group, including nurses (n = 8), doctors (n = 6), physiotherapists (n = 5), one medical laboratory technician, one occupational therapist, and one porter. The majority of the participants worked at hospitals in Capital which ties in well with the fact that the disease burden from COVID-19 was larger in Capital than in the rest of Denmark. On average the HCPs who were interviewed were 39.5 years old and had worked in their profession e.g., doctor or nurse for 7.3 years (Table 1). Their professional backgrounds and responsibilities varied. Most of them were not necessarily normally employed to care for or treat infectious patients but became part of the ward due to staff shortages and the urgency of the situation. The majority volunteered to sign up, while fewer were appointed by management and encouraged to participate based on their specific skill set. Due to the preponderance of similarities in the statements HCPs made during interviews, our analysis does not divide them by profession.

**Table 1:** Baseline characteristics of participants (n = 22)

Region	n (%)
Capital	14 (64)
Zealand	2 (9)
North Jutland	2 (9)
Central Jutland	4 (18)
Southern Denmark	0 (0)
**Sex**	**n (%)**
Female	12 (55)
Male	10 (45)
**Professional title**	**n (%)**
Nurse	8 (36)
Doctor	6 (27)
Physiotherapist	5 (23)
Medical laboratory technician	1 (4)
Occupational therapist	1 (4)
Porter	1 (4)
**Years in profession**	**n (%)**
2−10	9 (41)
11−20	10 (45)
21−44	3 (14)
**Specialty/Department**	**n (%)**
Intensive care	8 (36)
Accident and emergency	2 (9)
Infectious disease	1 (4)
Paediatrics	1 (4)
PhD student/Researcher	3 (14)
Gastrointestinal	1 (4)
Ear, nose, and throat	1 (4)
Pulmonology	2 (9)
Cardiology	1 (4)
Endocrinology	1 (4)

To achieve transparency in the production, interpretation, and presentation of data, we applied a reflexive methodology [14], which encompasses four levels of interpretation (Table 2), also known as quadri-hermeneutics, which allows reflexive interpretation when interpreting subjects and interpreting the interpretation [14].

**Table 2:** Levels of interpretation in reflexive methodology

Level of interpretation	Activity
First	Data collection and production
Second	Data analysis: empirically founded interpretation
Third	Data analysis: critical and theoretical interpretation
Fourth	Researchers’ self-reflection on own production of knowledge

The next four sections will present how the four levels of interpretation were applied methodologically in our study.

### Data collection and production

Using a semi-structured interview guide (Table 3) four experienced qualitative researchers conducted separate interviews. To minimise bias to the extent possible while interviewing, the researchers used open questions, discriminating questions, and moments of silence to give the interviewee time to associate and reflect and to independently break the silence with meaningful information [13].

Ethically, due to the limitations imposed by COVID-19 restrictions, the interviews were conducted by telephone from May to September 2020 and lasted 25 min on average. Participants could decide when to schedule their interview, which was audio recorded and then transcribed verbatim by the interviewer or a research assistant as quickly as possible after the interview to improve recall. Data saturation occurred when no new concepts, categories, or dimensions emerged in our subsequent analysis.

**Table 3:** Semi-structured interview guide

Could you please tell me where you earned experience treating/caring for patients with COVID-19?
Could you please tell me how you started working with patients with COVID-19?
Did you voluntarily choose to work with patients with COVID-19?
Were you motivated to take on the task/Did you resist taking on the task?
What thoughts did you have before you embarked on the task/job?
How did you prepare for the task/job?
What does it mean for you to care for/treat patients with COVID-19 − both professionally and personally?
Did you miss your specialty or ward during the time you cared for/treated patients with COVID-19 − why and how?
How did you experience your professionalism being affected?
How did you experience your collaboration with co-workers during the period?
How did the interprofessional collaboration function on a practical level?
How did you experience working in isolation wearing personal protective equipment?
What was it like to communicate with patients while wearing personal protective equipment?
What thoughts have you had about contracting the virus? (At work/at home/in society)
How did your family and friends react when you were a frontline worker?
What does it mean to you that the COVID-19 pandemic is a worldwide challenge?
Has working with patients with COVID-19 affected your thoughts and plans − professionally and/or personally?
How do you think your local management/hospital management has handled COVID-19?

Using reflexive methodology [14], data collection, i.e. the production of data, represents the first level of interpretation, which in our case comprised interviewing 22 HCPs and transcribing the interviews. The interview guide, which focused on how the HCPs talked about their professionalism, both their own and the team’s skills and abilities, helped the interviewer to maintain focus while simultaneously providing an opportunity to improvise with follow-up questions based on the HCP responses. At this level, interpretation had already been initiated as both the interviewer and the HCP were engaging in a dialogue that involved follow-up questions based on (pre)understandings about the meaning of what was said. The interviewers, nonetheless, explored what was said as openly as possible throughout the interview.

### Data analysis

The second level of reflexive methodology involves interpretation of the transcribed interviews. Specific procedures alone cannot guarantee high quality interpretation, which also depends on how skilled the researcher is at identifying empirically founded themes that are also meaningful on a larger scale [14]. To identify reliable themes and to minimise selection bias, everyone on the research team read the interviews individually before collectively interpreting each interview and identifying strong units of meaning, which led to the identification of thirteen initial themes. After an additional read through units with a similar meaning were put into the same group. Three main themes were found, two of which were merged. This empirical data forms the basis of the study. To achieve a hermeneutic interpretation, the process moved from wholeness to individual parts, to recontextualisation, to a new wholeness [14], the wholeness produced constituting the empirical themes that provided a detailed understandings of how being in the frontline of COVID-19 affected learning and skills development and interprofessional collaboration among HCPs. The Findings section presents the results more specifically.

The third level comprises a critical interpretation of the themes, which involved contextualisation in relation to relevant research within the field of interprofessional care and collaboration, as well as theoretical conceptualisation. To gain an understanding of how being in the frontline of COVID-19 affected learning and skills development, we applied Dreyfus et al.’s [15] concept of intuition and human expertise, combined with Benner’s [16] added insights. According to this concept, adults acquire skills through five stages of learning: novice, advanced beginner, competent, proficient, and expert [16]. Novices, who generally need to follow well-defined and context-free rules, find being flexible difficult and may know-that, which refers to general theoretical knowledge and logic rules, but lack know-how, which refers to the ability to transform and adapt their knowledge. On the other end of the five stages of learning, experts, on the other hand, possess extensive know-how, i.e. tacit knowledge, and are not always consciously aware of their own skills and abilities, which means that their know-how is nearly invisible to them. In other words, experts see but do not always recognise that they see. Know-how nevertheless becomes visible in its absence, i.e. when practitioners encounter new situations that cause them to recognise that their know-how is inadequate for completing the task at hand. In those situations, experts actively reflect on the how their “knowing that” can be moulded, expanded, and used differently to provide new options for taking action in the situation [15, 16].

To gain insight into how being in the frontline of COVID-19 affected interprofessional collaboration among HCPs, we applied Wackerhausen’s [17] theory on professions and collaboration in the health field due to its theoretical framework, which critically examines interprofessional collaboration. According to Wackerhausen [17], genuine collaboration across professional boundaries involves crossing them to perform the task at hand, which ultimately expands the knowledge and skill level of the individual professions.

Genuine interprofessional collaboration, however, is rare because it often practiced superficially or even avoided. Even though what Wackerhausen [17] calls the caring and healing professions, e.g. nursing, medicine and physiotherapy, are united by the shared goal of doing what is best for the patient, they often practice external cooperation, with the various professions isolated, which leaves the status quo intact. Wackerhausen [17], who believes that interprofessional collaboration is seldom motivated interprofessional, states that some of the associated challenges involve not professionalism itself, but what goes on beyond or behind the professionalism, such as the norms, habits, and values of the profession, not to mention the underlying organisational framework of the embedded habits. To truly be a member of a profession, the individual must be socialised in a particular way of working, i.e. in what is done and what is not done. From this interprofessional standpoint, the individual professional understands other professions regarding the explicit division of work and various areas of responsibility, as well as the profession’s more implicit and embodied aspects, such as habitual ways of talking, explaining, valuing, and assuming, in addition to professional norms, habits, traditions, values, ​​and attitudes. Finally, overcoming these often implicit norms can be a challenge [17].

To reach the goal of gaining new understandings and analytical generalisability, abductive reasoning was applied and the cross analysis of the empirical data at the second level of interpretation was combined via contextualisation in relation to other research and theoretical conceptualisations. The Discussion section presents this level of interpretation in more detail. Finally, the fourth level of interpretation comprises the self-reflection of researchers on their own texts and claims to authority, including theoretical choices, opt-outs, and selection of voices represented in the text (Table 4) [14].

**Table 4:**
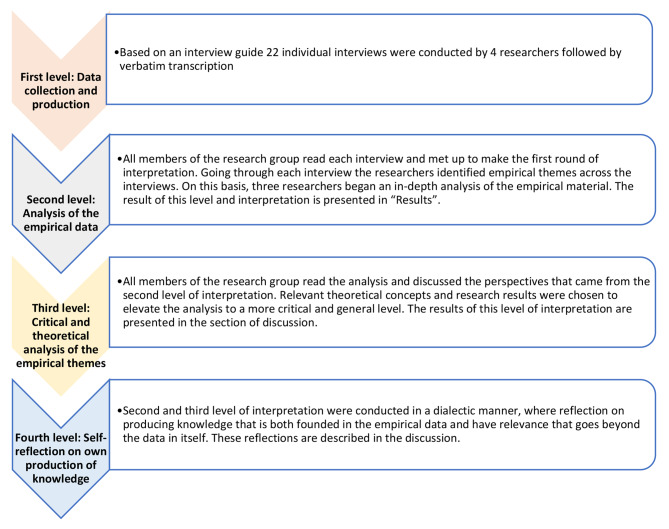
The process of qualitative data analysis

### Ethical considerations

Approved by the Danish Data Protection Agency (P-2020-276), the study was performed in accordance with National Research Ethics Committee guidelines published in the Danish Act on Research Ethics Review of Health Research Projects, as well as with the Declaration of Helsinki [18]. Prior to study participation, HCPs received information about the purpose of the study and their right to withdraw from it at any time without providing a reason. They also provided informed written consent, just as the researchers supplied information on how the data collected would be anonymised using identification codes to preserve confidentially.

## Results

In our exploration of the experiences of HCPs in the frontline of the COVID-19 pandemic, we learned more about how frontline work affects interprofessional collaboration during a pandemic and identified two overall themes: (1) into the unknown and (2) in the same boat, which will be analysed closely in the following.

### Theme 1: into the unknown

HCPs became involved in working in a COVID-19 ward in a variety of ways, some were picked by management and asked to join in the frontline work: “*I’ve worked on an emergency ward for six years (…), and I have to say that this was the reason why I was chosen to do it* [being part of the COVID-19 team] *by my supervisor*” (HCP 1). The HCPs felt that being carefully chosen provided professional recognition and they perceived it as a sign that they possessed the right skill set and knowledge to care for and treat patients with COVID-19, likely reinforcing their own belief that they could handle the task. A common experience for the HCPs in this study is that they all presented a keen sense of professionalism, most of them having volunteered for the task, with only a few selected by their supervisors and encouraged to participate, regardless of a weak desire to do so.

At the beginning of the pandemic, everyone involved embraced the unusual situation and accepted the temporary working conditions, throwing themselves into it while gathering all the courage they could: “*(…) It was a bit like being at war. You could say we were the frontline soldiers. We just had to do it*” (HCP 3). The gravity of the situation was clear to everyone, leaving no time to hesitate because they all played a vital role in the war. Thus, even though no one knew what they had signed up for, they described the situation as extraordinary, and they could not just: *“sit on the bench as substitutes*” (HCP 9). They showed a strong commitment and wanted to get involved in the new workflows, even though this meant they had to dexterously apply themselves to new, unfamiliar tasks.

The strong sense of personal accountability among HCPs to help was present regardless of their years of experience and skill level. Some HCPs were doctors, nurses, and physiotherapists who cared for and treat critically ill patients daily, while others had not had direct patient contact for several years. Nevertheless, the process of learning was the same for all of the HCPs involved, with everyone forced to acquire new skills due to the novelty of the tasks required. The HCPs dealt with this in various ways: “*The first few days there, I felt like a nursing student because I felt like I needed help with a lot of things, but once you were shown or told how to do it, you could recall what to do*” (HCP 24). In the beginning, they felt that every task was beyond their skills and abilities, but by continuing to take an active part and take responsibility by asking co-workers for advice, their motivation to help overshadowed their uncertainty. Others found that the situation was exceedingly demanding: “*The only thing the patients had in common was that they had COVID-19. On top of that, they had so many other things that we also had to deal with. In those situations, I personally felt I was too unexperienced*” (HCP 6). Being relocated from their ordinary jobs, where they were experienced practitioners, to a new ward was overwhelming since they could not apply their skills in the usual way or rely on habitual, everyday practices. Confronted with the inadequacies of feeling like a rookie, the HCPs suddenly became aware of the high level of risk involved for both them and the patients, which was coupled with the uncertainty of having to balance their new professional identity.

Several unknown factors, such as the severity of the virus and its possible impact on the community and health services in Denmark, affected the HCPs in the frontline. They describe how frightened they were due to the working conditions and situation in southern Europe: “*We were, in a way, terrified, because we followed the news on what was happening in Northern Italy, and what the conditions were, and how sick the patients were. It was a whole new disease scenario that we were unfamiliar with. We also didn’t know how sick it might make us (…). We only had numbers, data, and video clips from abroad*” (HCP 10). HCPs openly acknowledged their fears about patients rapidly becoming critically ill. Despite what they learned from international colleagues, they felt highly unsure about what to expect. They also felt anxious about the risk of lacking resources, not to mention being forced to prioritise patients due to a lack of equipment.

All these unknowns made them, to a greater or lesser degree, feel inexperienced and unable to deliver the level of care they felt professionally and morally obligated to provide. This led to a variety of reactions among the HCPs, one of them stating: “*Some people were definitely insecure about being there. Being in a place where something else is suddenly expected of you, that you might not be used to doing … One person comes to mind … at the end* [of a shift]. *She just sat on a chair because she simply didn’t know what she should do*” (HCP 1). Having to care for patients with a broad range of diseases and complex needs became too challenging for some frontline HCPs. The combination of work changes and uncertainty led to a paralysing sense of devastation among some of them, though some found it challenging that no one knew everything, but everyone learned something along the way. The accompanying uncertainty made them doubt and question their work, as well as whether or not what they did was good enough.

Despite their feelings of inadequacy and inexperience, they also described the situation as having a positive outcome. Gradually, once they had eased into their new tasks and acquired the knowledge necessary to execute them, they began taking pride in their achievements: “*It’s been an extremely good example of what professional reflection really means, and what the impact is of allowing yourself to reflect and ask stupid questions, as well as be open about your ignorance in a forum where people are all in the same situation*” (HCP 4). There was a strong sense of community and an understanding that everyone was in it together, which helped them overcome challenges and resulted in a sense of personal growth and greater confidence in their own resilience.

### Theme 2: in the same boat

Inundated by the repercussions of a new, unknown illness, including new ways of combining staff on teams and the establishment of work routines in new environments, created a sense of uncertainty but also made people feel that they were in the same boat. Redeploying staff and establishing new frameworks provided a foundation for making people feel that they were equals, that is, an interdependent community: “*(…) We were so different, very different backgrounds, came from so many different specialties, so you became really good at helping each other and being good at offering to do what you were especially good at. I think that the open-mindedness that was out there was cool. It felt like we were in the same boat*” (HCP 1). Although their level of experience and specialist knowledge varied, they regarded each other as equals and supported each other in the transition into an unknown area. There was a tacit understanding that everyone would constructively work together in their new interprofessional teams to meet the daunting, and perhaps unmanageable, task of treating patients with COIVD-19.

The new grouping of staff was initially perceived as a challenge, especially in emergency situations, as no one possessed deep familiarity with the applicability of their own skills in the current situation, let alone that of new co-workers, or even whether the staff had the requisite skills at all. This situation left the HCPs feeling uncertain about whether the others had their back. This lack of familiarity with one another’s co-workers, including their skill level, was difficult to cope with: “*Having to deal with so many new people and not quite knowing what your co-workers were capable of, if patients became deathly ill, was hard*” (HCP 1). As time passed and the HCPs settled into their new wards more, they found their place, making it easier for them to take advantage of their co-workers’ skills and abilities to solve tasks and challenges.

Often taken for granted, familiar co-workers represent an invaluable resource on a daily basis and were in short supply in the frontline setting. Being surrounded by new co-workers made the HCPs more mindful of the importance of the people they work with on a daily basis: “*(…) I’ve probably also become more aware of what it means to have close co-workers (…) The fact that you have each other’s backs, it really means something for one’s job satisfaction*” (HCP 1). The sense of togetherness the HCPs experienced with their new co-workers when working at the frontline battling COVID-19 was noticeable, just as their working relationship with new co-workers was deemed highly valuable, despite the forced, temporary, and somewhat artificial setup.

Since the situation at the beginning of the pandemic was so unique, with nothing resembling ordinary everyday life, a prevalent mindset became: “*One set aside the normal obstacles*” (HCP 12), with problems and challenges solved as they emerged. The time factor was important and respected across the various professions: “*People knew very well that we didn’t have time to fiddle around. So, it* [discussion and disagreement about minor issues] *was set aside a bit. There was, of course, some messes here and there. But they* [discussion and disagreement about minor issues] *were dealt with very quickly*” (HCP 12). The severity of the pandemic meant that there was a collective understanding of which tasks should be prioritised, thus, also which details required attention and what could be ignored. Moreover, there was no room for internal professional struggles: “*(…) We were all on an equal footing and we all played an important role*” (HCP 6). Many HCPs used the pronoun ‘we’ when emphasising the work being done during the pandemic, signalling that their current setup had.

clearly had an impact on how the various professions worked together, including the balance of power. Professional communities were undeniably necessary to treat patients with COVID-19.

## Discussion

Using a hermeneutic approach and reflective methodology, this study explored how being in the frontline of COVID-19 affected the learning and skills development of HCPs, as well as their interprofessional collaboration. In the following we discuss the two themes into the unknown and in the same boat, in light of learning theory, i.e. learning in practice, and theory on interprofessionalism.

### Methodological considerations

A strength of this study is the diversity of the participants, also geographically, number of years in their profession, and knowledge level. The HCPs were recruited through purposeful sampling and convenience sampling, which was the most relevant way to address their experiences and appropriately gain access to highly relevant, urgently needed data during a crisis. However, a disadvantage of this sampling method is that the participants may represent HCPs who like to participate and had the mental surplus to do so, while more skeptical or vulnerable HCPs did not participate, preventing their experiences from being analysed.

The study was conducted during and after the first wave of the pandemic in Denmark, which means the experiences and points that we highlight and describe may have changed over time, since the HCPs acquired more experience during the second wave of the pandemic. Due to the risk of virus transmission between HCPs and researchers, the interviews were carried by telephone, which precluded taking nonverbal communication into account. Regardless of this disadvantage, the HCPs were willing to participate in the study and share their experiences with the research team.

Applying a reflexive methodology allowed a systematic interpretation process, with an empirically driven analysis followed by theoretical conceptualisation. It is important to note, however, that there is always more than one way to interpret a text. The empirical themes, into the unknown and in the same boat, combined with the conceptualisation of developing skills in practice and interprofessional collaboration, provided a certain perspective of what was at stake on the COVID-19 frontline – leaving other perspectives ignored. Finally, it was beyond the scope of this study to examine the possible differences that existed among the professions since it centred on the collective subject, i.e. HCPs as a group.

#### Into the unknown—from expert to novice

The first wave of the COVID-19 pandemic pushed the healthcare system and HCPs to their capacity in a situation where everything was unfamiliar, largely incapacitating everyday practices and know-how, similar to Chandler-Jeanville et al.’s [19] findings. Although experienced practitioners, the HCPs described feeling like students again. Considering Benner’s [16] theoretical contribution to nursing in terms of learning and the five stages of how adults acquire skills, it is evident that the HCPs’ learning development underwent an educational process. When COVID-19 commenced, they were all experienced practitioners within a specific field, but were catapulted into an unknown field when they became frontline workers and were expected to deal with numerous new and unfamiliar tasks. Propelled into this situation, they rapidly underwent personal growth and their professional confidence grew. From a theoretical perspective [16], the HCPs went from being experts in their own fields to being novices in the field of COVID-19, but rather quickly built expertise in the treatment and care of patients with COVID-19, returning them to their position as experts once again.

It is of course not unusual that for practitioners placed in a different context or specialty to move from being an expert to experiencing themselves as being a novice [16]. The COVID-19 frontline differed, as *no one* was an expert in the treatment and care of patients with COVID-19, leaving a vacuum, with nobody to take on the role of the experienced supervisor for newcomers. Furthermore, no standardised guidelines for treatment and care had been developed at the time. The sparse knowledge available was based on the disease’s frightening progression in China and southern Europe [20]. The normal support structures in the healthcare system were simply non-existent. On top of the HCPs’ uncertainty about their skills and the absence of guidelines for practice was the overshadowing fear of contracting the disease [12].

This unknown territory, where not a single expert was to be found, only novices, not to mention the paucity of evidence-based knowledge, pushed the HCPs to explicitly reflect with each other, even though many of them had not known one another previously. Their tacit know-how was no longer adequate, with the general lack of knowing-that in terms of COVID-19 giving rise to constant and conscious reflection, which the HCPs also described as extraordinary and resulting from the unusually tolerant atmosphere and strong team spirit.

#### In the same boat—the value of interprofessional collaboration

In the newly created wards for patients with COVID-19, the HCPs comprised, e.g. nurses, doctors, physiotherapists, and occupational therapists from various specialties and functions, some of whom had volunteered to work on the frontline, while others had been forced. A diverse group in terms of knowledge, experience, attitudes, and skills, they also did not know each other beforehand. Despite this, the HCPs experienced that their collaboration was unusually good, where it became apparent that no single profession had the ability to manage the situation alone, which highlights the significance of interprofessional collaboration as an essential part of healthcare systems [21].

In light of how Wackerhausen [17] defines genuine collaboration, where no single profession can manage tasks without assistance, it can be said that the HCPs indeed had genuine interprofessional collaboration on the COVID-19 frontline. The HCPs described a unique atmosphere, where people were equals and interdependent, which indicates that the barriers that normally obstruct interprofessional collaboration were put aside. The standpoint of the interprofessional “we”, from which other professions are normally understood, was substituted by a different kind of “we”, namely the frontline workers as a group with a strong sense of togetherness.

Wacherhausen [17] asserts that overcoming the barriers embedded in a profession’s implicit and embodied norms, habits, traditions, values, ​​and attitudes on what “we” and “they” do and do not do is challenging, but the COVID-19 situation magnified the obvious necessity of genuine interprofessional collaboration. On the journey from expert to novice and back, the HCPs were humble about the knowledge they lacked but also recognised that they must work together. Thus, working on the pandemic frontline had a unifying effect on HCPs, who shared a feeling of not knowing and of seriousness that made it necessary to be professional together – not separately. Metaphorically, they were in the same boat, where genuine interprofessional collaboration kept the boat on an even keel in unsteady waters.

### Recommendations for interprofessional education and practice

This study provides new knowledge on frontline HCPs during the first wave of the COVID-19 pandemic in terms of learning and developing new skills, as well as the importance of interprofessional collaboration. Our results suggest a need for continuing to focus on the importance of reflection to create the opportunity for a well-functioning collaboration. This study shows that the HCPs, despite the influence of their professionalism, were able to acquire new expertise and expand on it in the COVID-19 wards. A major reason this was possible was due to the completely unique and genuine interprofessional collaboration that took place based on a sense of togetherness created through interprofessional reflection.

## Conclusion

This study shows that the HCPs in the frontline of COVID-19 were sent into unknown territory, where everybody was a novice, where no experts or evidence-based guidelines were available to inform practice. Tacit know-how was no longer adequate, and a general lack of knowing-that in terms of COVID-19 forced the HCPs to explicitly reflect with one another. The atmosphere on the frontline was unique, allowing everybody to work as equals and interdependently, the barriers normally obstructing interprofessional collaboration pushed aside. The intraprofessional “we” from which other professions are normally understood was substituted by a different kind of “we”, namely the frontline workers as a group with a strong sense of togetherness, resulting in genuine interprofessional collaboration. In conclusion, the main point is that the HCPs went from being experts in their own fields, to being novices in the frontline of COVID-19, and then back to being experts through interprofessional collaboration that included shared reflection.

## Data Availability

All authors have full access to the primary raw data (interview transcripts), which are in Danish and stored on a sensitive logged data file at the Copenhagen University Hospital as requested by the Danish Data Protection Agency. The authors can provide the journal with access to the raw data upon request. MH can also provide access to study data upon reasonable request.

## Abbreviations

COVID-19: Coronavirus disease 2019
HCP: Healthcare professionals
WHO: World Health Organization

## References

[1] Khaled Habas C, Nganwuchu F, Shahzad R, Gopalan M, Haque S, Rahman, Anwarul Azim Majumder & Talat Nasim. (2020) Resolution of coronavirus disease 2019 (COVID-19), Expert Review of Anti-infective Therapy, 18:12, 1201–1211, Available at: 10.1080/14787210.2020.1797487 10.1080/14787210.2020.1797487 32749914

[2] Danish Health Authority, F406EAA6DA81D6EA91A64F1A087. COVID-19 i Danmark – 23. marts 2020 [COVID-19 in Denmark – 23 March 2020]. Copenhagen, (2020), Available at: https://www.sst.dk/-/media/Nyheder/2020/COVID-19-i-Danmark_-Epidemiens-foerste-boelge_-Status-og-Strategi_-Version-23_-marts-2020.ashx?la=da&hash=263A3D8EAB851F406EAA6DA81D6EA91A64F1A087

[3] Danish Health Authority. Status på COVID-19 ved indgangen til den tredje uge af epidemiens første bølge i Danmark, med særligt fokus på intensiv kapacitet [Status of COVID-19 at the start of the third week of the first wave of the epidemic in Denmark, with a special focus on int]. Copenhagen, Available at: https://www.sst.dk/-/media/Nyheder/2020/Status-p-COVID19-ved-indgangen-til-den-tredje-uge-af-epidemiens-frste-blge-i-Danmark-med-srligt-foku.ashx?la=da&hash=1069D4E11552B76484FA56EAD1D20A7DF8E79BC7.

[4] Danish Health Authority. Notat om reduktion af hospitalsaktivitet ifm COVID-19 [Note on reduction of hospital activity in terms of COVID-19]. *Sagsnr. 04-0101-35*. Avaliable at: https://www.sst.dk/-/media/Udgivelser/2020/Corona/Hospitalskapacitet/Notat-om-reduktion-af-hospitalsaktivitet-ifm-med-COVID-19.ashx?la=da&hash=C2EF7016267E5DF9250BD0568276A6792775A73E.

[5] Danish Health Authority. The Danish healthcare system. Available at: https://www.sst.dk/-/media/Udgivelser/2017/Det-danske-sundhedsv%C3%A6sen/Det-Danske-Sundhedsv%C3%A6sen,-d-,-Engelsk.ashx

[6] Bou-Karroum L, El-Harakeh A, Kassamany Id I, Ismail H, Arnaout N, El, Charide R et al. Health care workers in conflict and post-conflict settings: Systematic mapping of the evidence. 2020 [cited 2021 Aug 5]; Available at: 10.1371/journal.pone.0233757 10.1371/journal.pone.0233757 PMC7259645 32470071

[7] Zhou Y, Zhang Z, Wang B, Ren G, Qi H, Wang X. Construction time, cost and testing data of a prefabricated isolation medical unit for COVID-19. Data Br [Internet]. 2020 [cited 2021 Dec 29];32. Available at: 10.1016/j.dib.2020.106068 10.1016/j.dib.2020.106068 PMC7380239 32775578

[8] Wee LE, Fan EMP, Heng R, Ang SY, Chiang JL, Tan TT, et al. Construction of a container isolation ward: a rapidly scalable modular approach to expand isolation capacity during the coronavirus disease 2019 (COVID-19) pandemic. Volume 42. Infection Control & Hospital Epidemiology. Cambridge University Press;; 2021. pp. 1162–4. 9. 10.1017/ice.2020.1222 PMC7562929 32962768

[9] Dall Jensen R, Bie M, Plønd Gundsø A, Martin Schmid J, Juelsgaard J, Louise Gamborg M et al. Preparing an orthopedic department for COVID-19. Acta Orthop. 2020;91(6):644–9. Available at: https://www.tandfonline.com/action/journalInformation?journalCode=iort20 10.1080/17453674.2020.1817305 PMC8023962 32907437

[10] Dolan B. Transition into a new job, FEMS Microbiology Letters, Volume 366, Issue 5, March 2019, fnz045. Available at: 10.1093/femsle/fnz045 10.1093/femsle/fnz045 30806657

[11] Richmond A, Cooper N, Gay S, Atiomo W, Patel R. The student is key: A realist review of educational interventions to develop analytical and non-analytical clinical reasoning ability. Med Educ 2020 Aug;54(8):709–19. Available at: 10.1111/medu.14137 10.1111/medu.14137 32083744

[12] Dagyaran I, Risom SS, Berg SK et al. Like soldiers on the front – a qualitative study understanding the frontline healthcare professionals’ experience of treating and caring for patients with COVID-19. BMC Health Serv Res 21, 666 (2021). Available at: 10.1186/s12913-021-06637-4 10.1186/s12913-021-06637-4 PMC8260234 34229686

[13] Polit DF, Beck CT. Nursing research : generating and assessing evidence for nursing practice. 11th ed. Wolters Kluwer; 2020.

[14] Alvesson M, Skölberg K. Reflexive methodology - new Vistas for qualitative research. Second edi. SAGE Publications Ltd; 2009.

[15] Dreyfus HL, Dreyfus SE, Athanasiou T. Mind over machine : the power of human intuition and expertise in the era of the computer;1986.

[16] Benner P. From novice to Expert: Excellence and Power in clinical nursing practice. Pearson Education (Us); 2001.

[17] Wackerhausen S. Collaboration, professional identity and reflection across boundaries, J Interprof Care, 23:5, 455–73, 10.1080/13561820902921720 10.1080/13561820902921720 19657938

[18] Inc WMA. Declaration of Helsinki. Ethical principles for medical research involving human subjects. J Indian Med Assoc. 2009 Jun;107(6):403–5. 19886379

[19] Chandler-Jeanville S, Nohra RG, Loizeau V et al. Perceptions and Experiences of the COVID-19 Pandemic amongst Frontline Nurses and Their Relatives in France in Six Paradoxes: A Qualitative Study. Int J Environ Res Public Health. 2021 Jun 29;18(13):6977. Available at: 10.3390/ijerph18136977 10.3390/ijerph18136977 PMC8297294 34209931

[20] Jiang F, Deng L, Zhang L et al. Review of the Clinical Characteristics of Coronavirus Disease 2019 (COVID-19): 2020. 10.1007/s11606-020-05762-w PMC7088708 32133578

[21] Lackie K, Najjar G, El-Awaisi A, Frost J, Green C, Langlois S, Lising D, Pfeifle AL, Ward H, Xyrichis A, Khalili H. Interprofessional education and collaborative practice research during the COVID-19 pandemic: Considerations to advance the field. J Interprof Care 2020 Sep-Oct;34(5):583–6. Available at: 10.1080/13561820.2020.1807481 10.1080/13561820.2020.1807481 32838595

